# Commentary: Evolution of chordal techniques for mitral valve repair

**DOI:** 10.1016/j.xjtc.2023.08.018

**Published:** 2023-09-01

**Authors:** J. Scott Rankin

**Affiliations:** Department of Cardiovascular and Thoracic Surgery, West Virginia University, Morgantown, WVa

**Figure:**
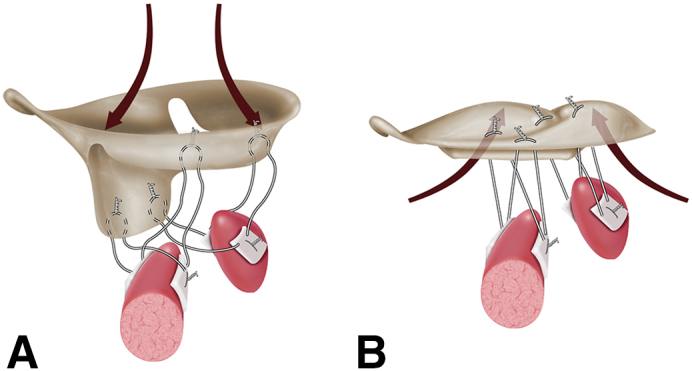
Diastolic (A) and systolic (B) appearance of a Barlow's valve after PTFE artificial chordal replacement.

**Central Message**

Developing techniques of chordal reconstruction was integral to mitral repair. Current methods and the history of these techniques is discussed.

See Article page 73.

In the 1960s and '70s, mitral valve replacement for mitral regurgitation was among the highest-risk procedures in cardiac surgery.^1^ Fifty years later, surgery for mitral regurgitation has become extremely safe and effective^2^—arguably among the major success stories in medicine. This transition was due largely to the development of contemporary mitral valve repair, and in this issue, Rehman and colleagues^3^ present their current technique for robotic artificial chord implantation in a highly lucid video. The purpose of this Commentary is to discuss some of the developmental steps that culminated in current repair efficacy.

Although many surgeons reported repairing mitral valves in the 1960s,^4^ it was Carpentier who developed the concept of mitral ring annuloplasty that is the basis of current reconstruction. To quote Carpentier^5^:

Prosthetic rings of a suitable shape and size are necessary to perform a measured annuloplasty which will [reproducibly] restore the normal [geometric] contour—and thereby both a normal orifice area and optimum function of the valve.

We forget now that mitral repair was not immediately accepted, and in fact, some of Carpentier's initial approaches, such as chordal shortening (Figure 1, *A*), were associated with an incidence of repair failure (Figure 1, *B*).^6^ After presenting such a case in morbidity conference in the 1980s, the author was intensely criticized by fellow faculty members for embarking on a new and unproven program. One often thinks about calling these colleagues now to say, “You see, mitral repair did work out.” But alas, they have departed.

**Figure 1:**
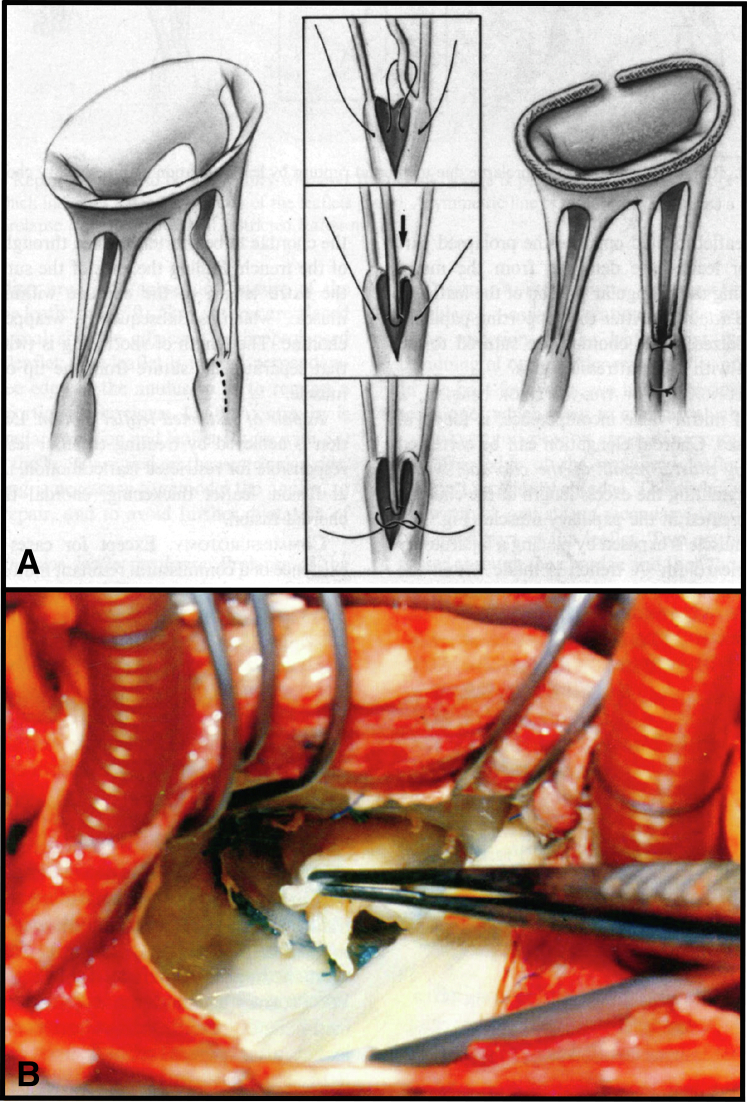
A, Carpentier's method for shortening elongated chordae. B, The method was associated in some patients with late chordal rupture.^5^^,^^6^

Toward the end of the 1980s, Frater and Vetter^7^ addressed the chordal support problem by inserting polytetrafluoroethylene sutures for artificial chordal replacement (ACR). Frater had worked with Ellis on mitral repair while at Mayo Clinic,^8^ and in a 1962 *Lancet* article, he made the prophetic statement: “…the patient with a mitral prothesis is a patient for life.”^9^ His advent of ACR contributed significantly to transitioning away from replacement, and as he later stated^10^:

The initial growth [of ACR] was entirely without promotion, sponsorship, or marketing—driven by the desire of the individual surgeon to achieve the fundamental goals of restoring valve competence and the modern reality of mitral valve repair.

Many surgeons made important contributions to ACR.^11^, ^12^, ^13^, ^14^, ^15^, ^16^ In early clinical experience, it became evident that achieving precise chordal length was critical. By 1995, we began tying an initial temporary knot, then completing the chordal length adjustment after ring insertion. Initial submission of this technique for publication was met with an interesting, if not amusing, review: “…the originality, the scientific accuracy, the relevance and the presentation are really very poor and I do not even know where to start with my remarks and suggestions to try to improve this stuff. Personally, I would recommend outright rejection of this manuscript.” However, with some effort, the technique of adjustable ACR was published,^17^ followed by 2 more articles.^18^^,^^19^ At the Society of Thoracic Surgeons meetings 20 years ago, we presented videos of this approach (Videos 1 and 2), and also posted on CTSNet. ACR proved especially useful for repair of anterior leaflet prolapse,^20^ and 15 years ago, a transition was made to implanting chords robotically (Video 3),^21^ a progression that ultimately culminated in the current highly effective techniques for robotic mitral repair, as nicely illustrated in Rehman and colleagues' video.^3^ Even now, it is common to be contacted by patients who are 25 to 30 years post-ACR who have no subsequent valve problems. Such is the goal of cardiac valve repair.

## Conflict of Interest Statement

Dr Rankin is a consultant for BioStable Science and Engineering Inc, Austin, Tex.

The *Journal* policy requires editors and reviewers to disclose conflicts of interest and to decline handling or reviewing manuscripts for which they may have a conflict of interest. The editors and reviewers of this article have no conflicts of interest.

## Supplementary Data

Video 1 Mitral valve repair for Barlow's syndrome using adjustable artificial chordal replacement. Presented at the Society of Thoracic Surgeons meeting 2004 and posted on CTSNet. Video available at: https://www.jtcvs.org/article/S2666-2507(23)00294-8/fulltext.

Video 2 Adjustable artificial chordal replacement (ACR) for repair of mitral regurgitation due to isolated posterior leaflet prolapse. Presented at the Society of Thoracic Surgeons meeting 2005 and posted on CTSNet. Video available at: https://www.jtcvs.org/article/S2666-2507(23)00294-8/fulltext.

Video 3 Robotic artificial chordal replacement for repair of mitral valve prolapse. Presented at the International Society for Minimally Invasive Cardiothoracic Surgery meeting 2008. Video available at: https://www.jtcvs.org/article/S2666-2507(23)00294-8/fulltext.
