# Supplementary figures and images for: Commentary: Evolution of chordal techniques for mitral valve repair
Source: JTCVS Tech. 2023 Sep 1;22:75–7. doi: 10.1016/j.xjtc.2023.08.018 (PMC10750497; doi:10.1016/j.xjtc.2023.08.018)

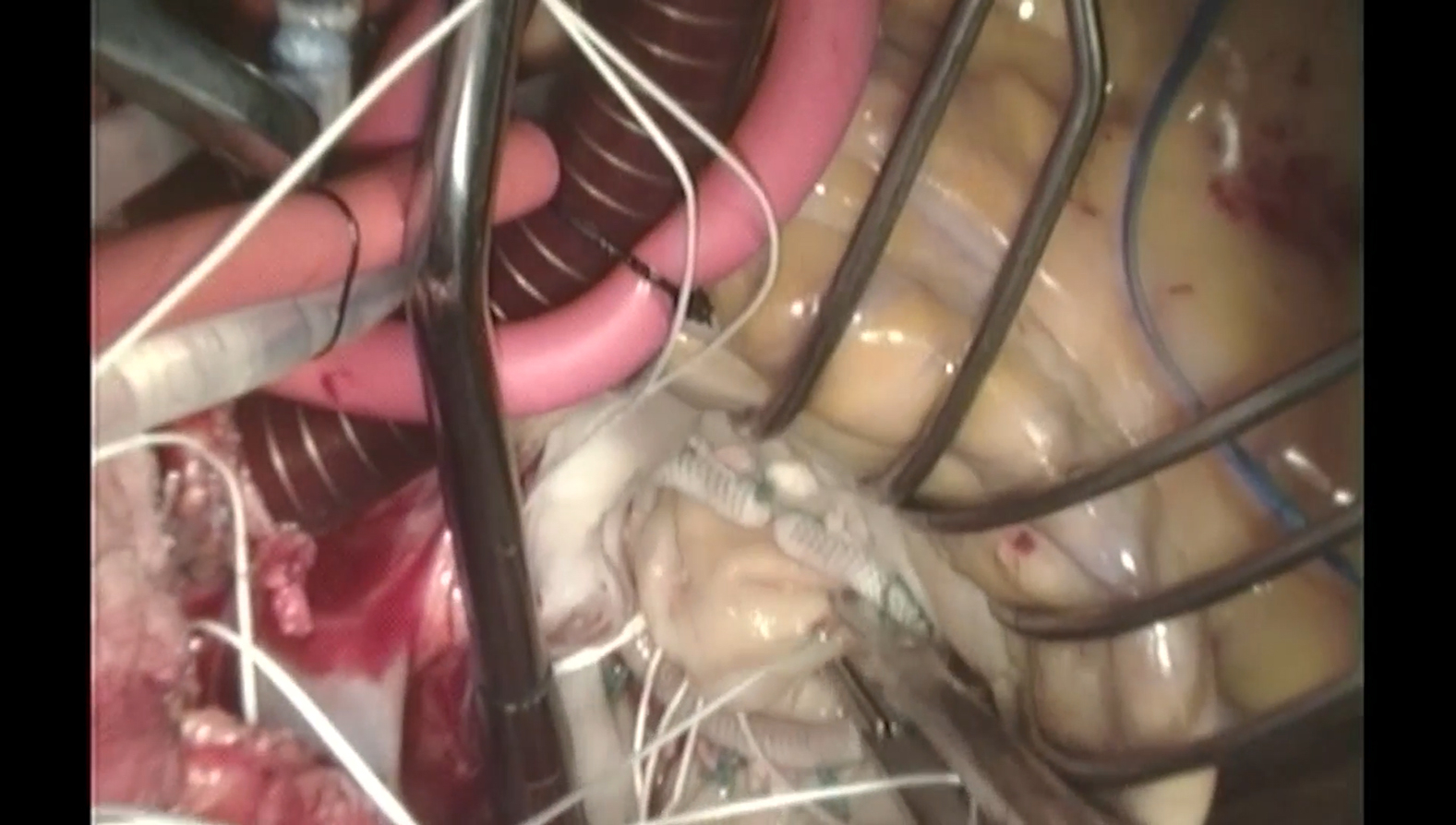

Supplement: Video 1 — Mitral valve repair for Barlow's syndrome using adjustable artificial chordal replacement. Presented at the Society of Thoracic Surgeons meeting 2004 and posted on CTSNet. Video available at: https://www.jtcvs.org/article/S2666-2507(23)00294-8/fulltext. [file fx2.jpg]

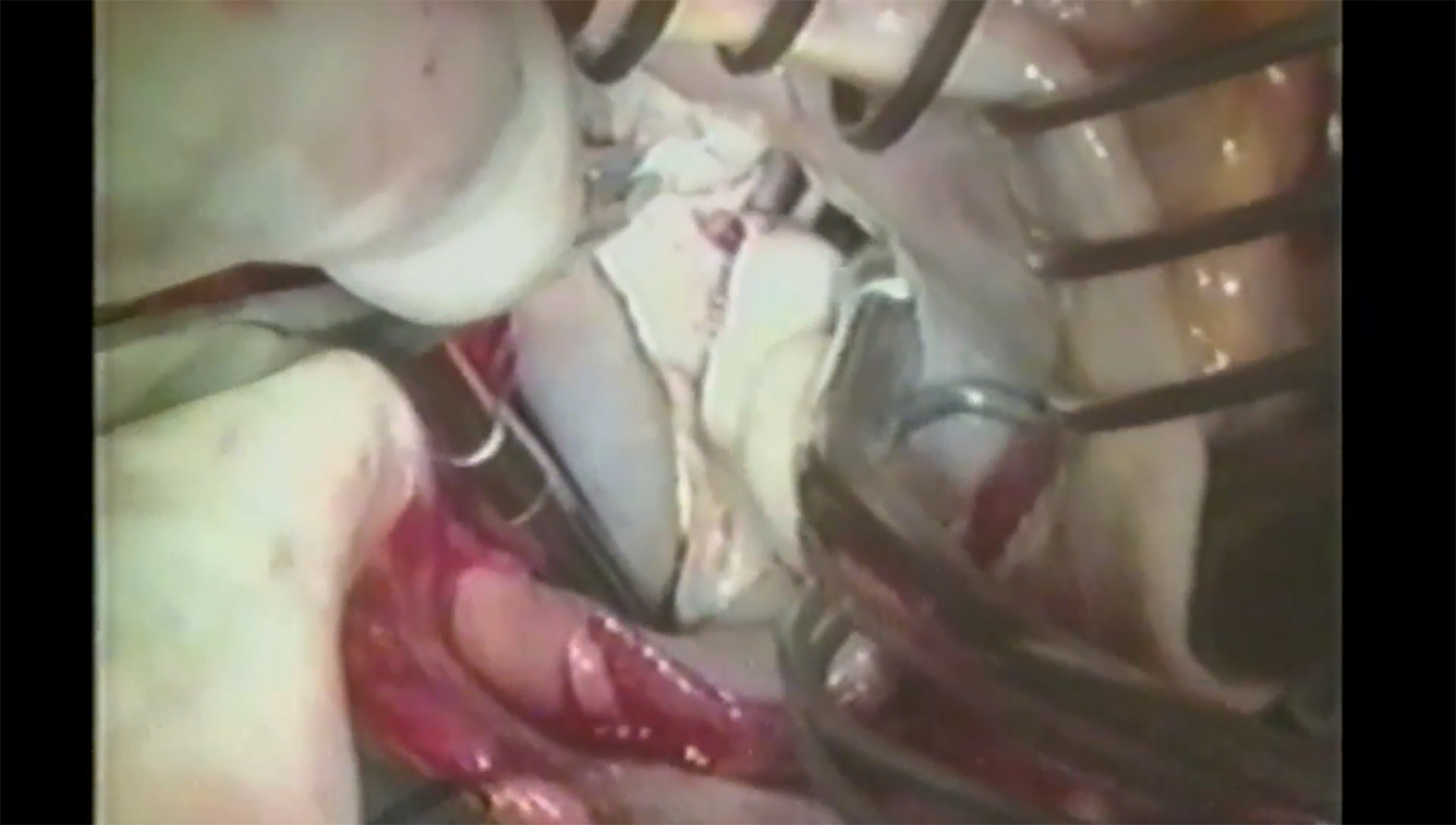

Supplement: Video 2 — Adjustable artificial chordal replacement (ACR) for repair of mitral regurgitation due to isolated posterior leaflet prolapse. Presented at the Society of Thoracic Surgeons meeting 2005 and posted on CTSNet. Video available at: https://www.jtcvs.org/article/S2666-2507(23)00294-8/fulltext. [file fx3.jpg]

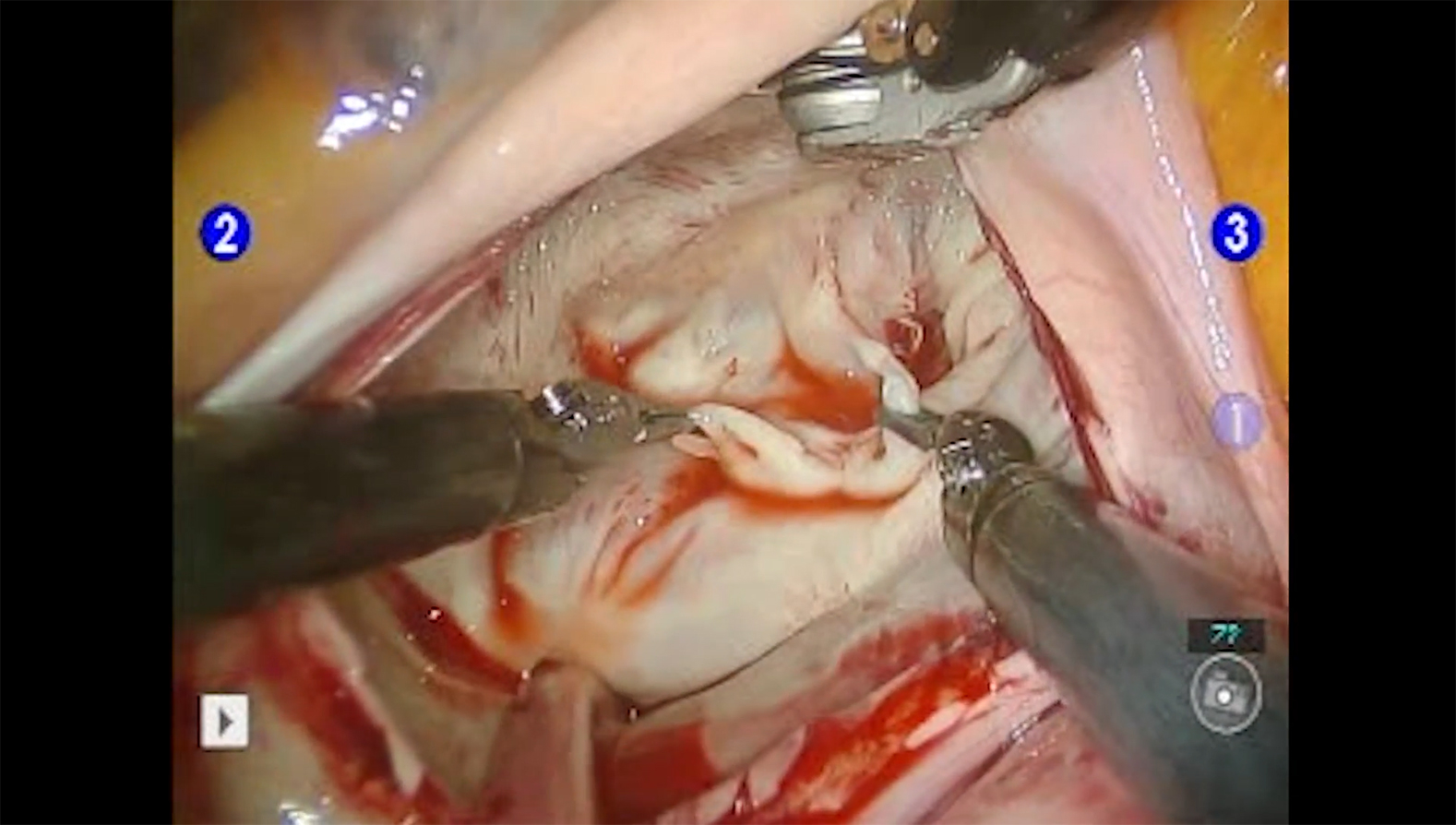

Supplement: Video 3 — Robotic artificial chordal replacement for repair of mitral valve prolapse. Presented at the International Society for Minimally Invasive Cardiothoracic Surgery meeting 2008. Video available at: https://www.jtcvs.org/article/S2666-2507(23)00294-8/fulltext. [file fx4.jpg]
